# Innovations in Chewable Formulations: The Novelty and Applications of 3D Printing in Drug Product Design

**DOI:** 10.3390/pharmaceutics14081732

**Published:** 2022-08-18

**Authors:** Lucía Rodríguez-Pombo, Atheer Awad, Abdul W. Basit, Carmen Alvarez-Lorenzo, Alvaro Goyanes

**Affiliations:** 1 Departamento de Farmacología, Farmacia y Tecnología Farmacéutica, I+D Farma (GI-1645), Facultad de Farmacia, Materials Institute iMATUS and Health Research Institute of Santiago de Compostela (IDIS), Universidade de Santiago de Compostela, 15782 Santiago de Compostela, Spain; 2 Department of Pharmaceutics, UCL School of Pharmacy, University College London, 29-39 Brunswick Square, London WC1N 1AX, UK; 3 FabRx Ltd., Henwood House, Henwood, Ashford, Kent TN24 8DH, UK

**Keywords:** 3D printed medicines and pharmaceuticals, orally disintegrating formulations, human and veterinary medicine, precision medicine, taste masking, digital healthcare, chewable oral formulations, patient acceptability and palatability, orodispersible tablets and films

## Abstract

Since their introduction, chewable dosage forms have gained traction due to their ability to facilitate swallowing, especially in paediatric, geriatric and dysphagia patients. Their benefits stretch beyond human use to also include veterinary applications, improving administration and palatability in different animal species. Despite their advantages, current chewable formulations do not account for individualised dosing and palatability preferences. In light of this, three-dimensional (3D) printing, and in particular the semi-solid extrusion technology, has been suggested as a novel manufacturing method for producing customised chewable dosage forms. This advanced approach offers flexibility for selecting patient-specific doses, excipients, and organoleptic properties, which are critical for ensuring efficacy, safety and adherence to the treatment. This review provides an overview of the latest advancements in chewable dosage forms for human and veterinary use, highlighting the motivations behind their use and covering formulation considerations, as well as regulatory aspects.

## 1. Introduction

The oral route is the most common route for administrating medicines as it is the most convenient and is easy to handle, making it the first choice for clinicians and most patients [1]. In general, oral formulations are considered to be cheaper than formulations designed for other routes [2]. Moreover, many drugs are well suited to be administered orally using different types of dosage forms, including liquids, capsules, tablets or chewable formulations.

**Figure 1:**
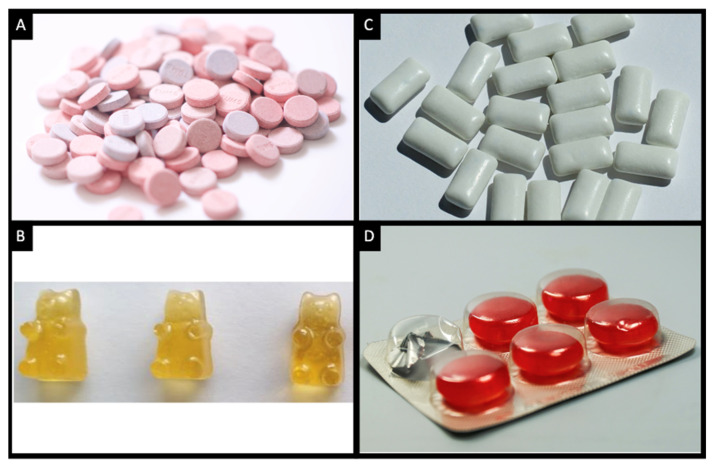
Images of (**A**) chewable tablets [7]; (**B**) chewable gummies [8]; (**C**) chewing gums [9]; and (**D**) lozenges [10]. All images were reprinted with permission from their original sources.

Despite their advantages, conventional solid (e.g., tablets and capsules) and liquid (e.g., solutions and suspensions) dosage forms still have some limitations [3,4,5]. One of the main disadvantages associated with the solid forms is the swallowing difficulties encountered by some patient populations (e.g., paediatrics and geriatrics) [4,6]. Although liquid dosage forms are easy to swallow, they suffer from stability issues and dosing errors [3]. Chewable formulations—e.g., chewable tablets, gummies, gums and lozenges (Figure 1)—on the other hand, are gaining attention due to their ease of administration, safety and lack of stability challenges. These formulations can be produced using different pharmaceutical methods, depending on the type of dosage form being made. However, most of these processes are complex, involving multiple unit operations.

Three-dimensional (3D) printing is an additive manufacturing tool that offers a sophisticated way of creating personalised chewable formulations [11]. The technology has been widely investigated to fabricate various types of 3D printed dosage forms, termed Printlets^TM^, in different sizes, shapes, flavours and drug doses [12,13,14,15]. Moreover, it offers the possibility of engineering multi-drug dosage forms, known as PolyPrintlets, which could benefit patients on a polypharmacy and simplify their dosing regimen [16,17,18]. This is achieved through the development of patient-friendly formulations that are tailored to each patient’s needs and preferences, improving medication adherence [19,20]. The benefits are particularly significant in the case of drugs with narrow therapeutic indices, where a small variation in the drug dose can cause severe side effects.

This review aims to provide an overview of chewable formulations, covering the most common approaches and formulation development processes used for their production. Particular attention is paid to the recent innovations made using 3D printing, highlighting its potential for solving technical issues and organoleptic properties, which are critical for efficacy, safety and adherence to treatment. Finally, regulatory aspects of chewable tablets are addressed. The searching criterion used to gather all the information is included as Supplementary Material (File S1: Literature searching criterion).

## 2. Advantages and Disadvantages

One main advantage of chewable tablets is their suitability to be administered to patients with swallowing difficulties, such as geriatric and paediatric patients and those suffering from dysphagia [21,22], improving their acceptability to treatments [23]. Another benefit is the absence of the need to co-administer them with water, making their use convenient for patient intake. Additionally, as they disintegrate in the mouth, part of the drug dissolves in the saliva and is consequently absorbed through the buccal cavity, avoiding the first-pass effect and increasing the drug’s bioavailability. Moreover, chewable tablets are not constrained by size, as they are designed to be chewed before they are swallowed.

In terms of disadvantages, it may be challenging to load chewable formulations with drugs having unpleasant or pungent tastes (e.g., bitter taste) without the addition of large amounts of sweeteners and flavouring agents. Chewable tablets are also hygroscopic and thus, must be stored in a dry place in airtight containers. Moreover, these formulations have been reportedly associated with incidents involving tooth damage or denture breakage resulting from excessive tablet hardness and oesophageal irritation.

## 3. Target Population for Chewable Tablets

Before prescribing a medication, a clinician must consider whether or not the patient can swallow it completely, safely and comfortably [24]. Some patient groups may have different requirements compared to the general population due to the inherent properties of each population. Thus, it is vital to select the best treatment that meets the special requirements (e.g., taste preferences, swallowing abilities, dose etc.) of each patient group. Patient acceptability to a pharmaceutical dosage form is critical for adherence and ensuring therapeutic outcomes are being met, especially in paediatric and geriatric populations [23]. Understanding patient adherence often involves an interplay of many factors that influence whether or not a patient successfully follows recommendations or completes a therapeutic program [22]. This includes the site of application, dosage form, composition of the formulation and the route of administration [24]. It has been reported that 1 in 11 primary care patients experience frequent difficulties in swallowing tablets and capsules, which is an ongoing problem that is highly disregarded by healthcare professionals [6]. It is therefore important for physicians to pay closer attention to swallowing difficulties to avoid non-adherence and inappropriate drug modifications, particularly in paediatric, geriatrics and dysphagia patients.

### 3.1. Dysphagia

Dysphagia refers to the difficulty in swallowing solids or liquids and includes any form of disruption to the swallowing process [25]. It has many different aetiologies and can affect a person of any age [26,27]. In general, people with anatomical or physiologic deficits in the mouth, pharynx, larynx and oesophagus may demonstrate signs and symptoms of dysphagia [25]. This condition may develop during infancy, childhood and adolescence due to congenital causes, acute infectious causes, injury, and neurodevelopmental delay [26]. In the middle-aged population, dysphagia could manifest from gastroesophageal and immunologic causes, which are predominantly associated with reflux, whereas in elderly patients, neurologic and oncologic causes are prevalent [26]. Age-related changes in swallowing physiology, as well as age-related diseases, are predisposing factors for dysphagia in the elderly [25]. Moreover, dysphagia contributes to a variety of negative health status changes, most notably, the increased risk of malnutrition and pneumonia [25].

Dysphagia not only affects the intake of food and drinks but also that of medicines [21]. Swallowing tablets or capsules can be problematic for this patient group, requiring modification of the formulation (e.g., crushing tablets or opening capsules) to facilitate administration [28,29,30]. However, this is associated with risks of altering the drug’s pharmacokinetic profile or its therapeutic activity, potentially leading to adverse effects due to dose dumping [31,32].

In this regard, chewable tablets could provide a suitable alternative to conventional oral dosage forms, which would greatly benefit patients by facilitating the passage of the dosage form into the digestive tract through the chewing process that precedes swallowing.

### 3.2. Geriatric Population

Typically, with ageing, patients may find swallowing a tablet uncomfortable, impacting their adherence to treatment, and deterring them from taking their medication(s). The implications become more pronounced in the case of patients on a polypharmacy (i.e., the regular use of five or more medications per day), leading to an increase in morbidity and mortality rates. Thus, it has become common practice for patients or their caregivers to manipulate a medicine to facilitate its administration. However, this is associated with a high risk for medication errors, resulting in dose variation or dose dumping (e.g., in the case of enteric-coated tablets). Alternatively, in some cases, medicines for other routes of administration could be repurposed for oral use. As an example, the content of ampoules for parenteral use could be administered perorally, given that the drug substance is stable in the gastrointestinal (GI) tract and that its peroral bioavailability is well understood [24]. Taking this into account, chewable tablets are considered suitable for use by elderly patients.

### 3.3. Paediatric Population

For decades, children have been regarded as “therapeutic orphans” [33] because pharmaceutical research, regulation and formulation development have been mainly focused on adults [34]. Thus, it has become common practice to modify dosage forms designed for adult administration before being given to children, either by preparing a suitable unlicensed medicine or by manipulating dosage forms at the point-of-care [32,35,36]. The use of unlicensed and off-label medicines (i.e., those prescribed and/or administered outside the terms of their marketing authorisation) is common in children due to their exclusion from trials during the drug development process [37].

In general, the physiological characteristics of paediatric patients rapidly change over time, making it a very heterogeneous population. It is well-known that children have different needs compared to adults and these differences have a huge impact on pharmacokinetics. Therefore, child-appropriate formulations with precise dosing are needed for efficient and safe therapy [38,39]. Due to this, efforts have been made in the EU and US to highlight this problem and find solutions to overcome existing gaps in paediatric treatments. Indeed, the Paediatric Committee at the European Medicines Agency (EMA) was established in 2007 and, together with the mandatory Paediatric Investigation Plan (PIP), forms the pillar of current regulations. The “Guideline on Pharmaceutical Development of Medicines for Paediatric Use” released by the EMA provides formulation criteria [40]. Whilst some of the defined attributes are the same as those for adult patients, there are significant differences and challenges that must be taken into account (e.g., heterogeneity, precise and appropriate dosing, swallowing difficulties, palatability and acceptability, and excipient safety) [24,40].

With these regulations in place, manufacturers have the opportunity to develop age-appropriate formulations that are both safe and efficacious. This should also facilitate carrying out clinical trials in children, enabling obtaining marketing authorisation for the use of these medicines in the paediatric population [35].

## 4. Types of Chewable Formulations and Conventional Manufacturing Methods

Chewable dosage forms could show different physical and mechanical characteristics, but all of them must be chewed to exert their intended action. Each type of chewable formulation and its manufacturing processes are described in detail in the next subsections.

### 4.1. Chewable Tablets

According to the United States Pharmacopoeia (USP), chewable tablets are oral dosage forms intended to be chewed and then swallowed by the patient rather than swallowed whole [41]. The USP differentiates two types of chewable tablets: those that may be chewed for ease of administration and those that must be chewed or crushed before swallowing to avoid choking and/or to ensure the release of the active ingredient [41]. The Japanese Pharmacopoeia defines chewable tablets as “tablets which are administered by chewing”, while for the European Pharmacopoeia (EP), chewable tablets “are intended to be chewed before being swallowed”.

In general, chewable tablets have a smooth texture, offer a pleasant taste and, ideally, should not leave a bitter or pungent aftertaste [42,43]. These dosage forms combine the advantages of conventional tablets in terms of manufacturability, dosing accuracy, portability and long-term stability [42], whilst providing favourable organoleptic and administration benefits. Chewable tablets may be preferred over conventional tablets and capsules when the required dose is high and the dosage form would be too big to pass through the oesophagus. They should be designed to be palatable and easy to chew and swallow. This is a useful patient-centric advantage, which can improve adherence to treatment, especially in patients who are unable or reluctant to swallow intact tablets or capsules due to their size or because of a disease condition [24,44,45,46].

The development of a successful formulation depends on selecting appropriate excipients. Many of the excipients used to prepare chewable tablets are similar to those used in conventional tablets (Table 1). The key excipients in chewable tablets include flavouring agents and sweeteners because they are intended to be chewed, and it is necessary to mask unpleasant tastes.

**Figure 2:**
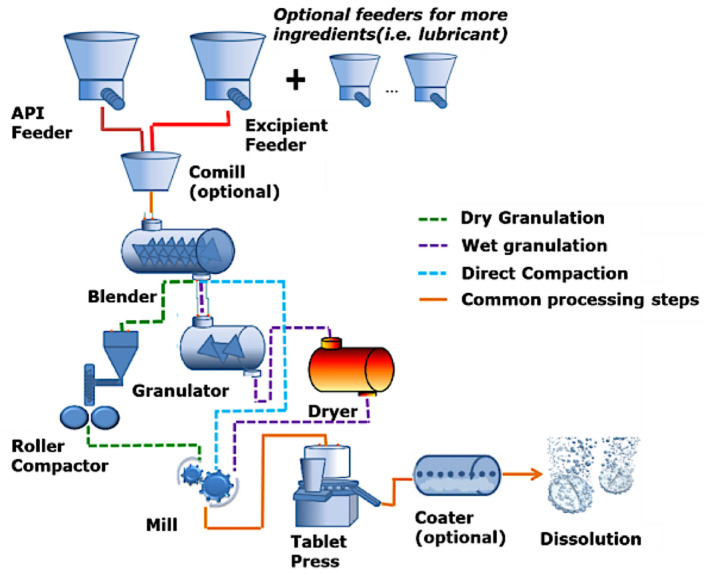
Schematic representation of dry granulation, wet granulation and direct compression. Reprinted with permission from [54].

Like other types of tablets, conventional manufacturing methods, such as wet or dry granulation [47,48] and direct compression [49], are used for the preparation of chewable tablets (Figure 2). The main advantages of using direct compression are its lower costs due to the fewer steps involved, the absence of a drying step, its suitability for moisture- and heat-sensitive drugs, and lower chances for microbial growth or cross-contamination [50,51]. Nonetheless, this production method has some limitations, such as being prone to segregation, can include limited drug content, and the poor compressibility of some substances [50]. In fact, it has been estimated that less than 20% of pharmaceutical materials can be directly compressed into tablets due to a lack of flow, cohesion properties and lubrication [52]. Therefore, materials must be blended with other directly compressible ingredients (e.g., α-lactose monohydrate or microcrystalline cellulose), or the powder must be granulated prior to compression to obtain flow and cohesion properties suitable for compression [50,52,53]. This increases the complexity of the process, making it more costly and laborious.

### 4.2. Chewing Gums

A chewing gum can be defined as a pliable preparation consisting of a gum base designed to be chewed and remains in the mouth rather than being swallowed [55]. Whilst in the mouth, this dosage form provides a slow and steady release of the drug contained inside it [56]. Therefore, the drug delivery process depends on the patient-dosage form interaction [57,58]. When the masticatory process is absent, no relevant drug release occurs. In fact, the controlled drug release action from the gum matrix is only active in the elastic state of the gum [57]. The masticatory activity of the patient determines the rate of transformation from the inactive, glassy, solid gum state to its active, rubbery, water-penetrating elastic mass responsible for regulating the drug release rate [57].

Medicated chewing gums consist of a masticatory gum core that may be coated. The core is composed of an aqueous insoluble gum base, which can be mixed with sweeteners and flavouring agents [59,60]. The coating can be applied as a film of polymers, waxes, sweeteners, flavouring agents and colourants or as a thick layer of sugar or sugar alcohols. The active ingredient may be present in the core, in the coating, or in both [59].

Medicated chewing gums have several advantages over other types of formulations [60]. They can be taken without water and can be administered discretely anywhere and at any time, releasing the drug and achieving the desired therapeutic activity over a suitable timeframe [60]. The gum can be removed by the patient, inhibiting the drug release at any time. Some diseases that affect the teeth or oral cavity can be treated or prevented (caries prevention and xerostomia) through local drug release in the mouth region [59]. According to the EP, chewing gums are not only intended for the local treatment of mouth diseases but may also be used for systemic drug delivery since drug absorption occurs through the buccal mucosa or in the GI tract. Drugs that are directly absorbed via the membranes lining the oral cavity bypass the first-pass effect and avoid degradation in the GI tract. On the other hand, drugs that are released but not absorbed in the oral cavity dissolve or disperse in the saliva and are swallowed down the gut [59].

Aspergum was the first medicated gum to be marketed in the US in 1924 [60]. It contains acetylsalicylic acid and is indicated as an analgesic and antipyretic agent [61]. Nowadays, the most common medicated gum that has had a great impact on the world is nicotine gum. It is indicated for the relief of nicotine withdrawal symptoms and to aid in smoking cessation [62,63,64,65]. Since their introduction, chewing gums have been well accepted by the general population [60], and efforts have been made to launch other active ingredients for different indications. This includes those for anti-caries or anti-plaque effect (e.g., fluoride [66,67,68], chlorhexidine [67,69,70], xylitol [67,71], sorbitol [71,72], enzymes [73], zirconium silicate [74] or zinc acetate [75]) [76,77,78], oral candidiasis (e.g., miconazole [79,80]), bacterial infections (e.g., combination of neomycin/gramicidin [81]) and fungal infections (e.g., nystatin [82]). Chewing gums containing caffeine have also been indicated for their systemic activity in alleviating the effects of insomnia or fatigue [83], sleep inertia [84] and improvement of the alert state [85]. Similarly, dimenhydrinate has been formulated in chewing gums for the treatment of motion sickness [86,87]. It is well absorbed after oral administration but undergoes first-pass metabolism [87]. Therefore, formulating it as a chewing gum could benefit from buccal absorption, improving its bioavailability and providing a faster therapeutic effect [88].

Methods used to manufacture chewing gums can be broken down into three main techniques [89].

#### 4.2.1. Conventional or Fusion Method

**Figure 3:**
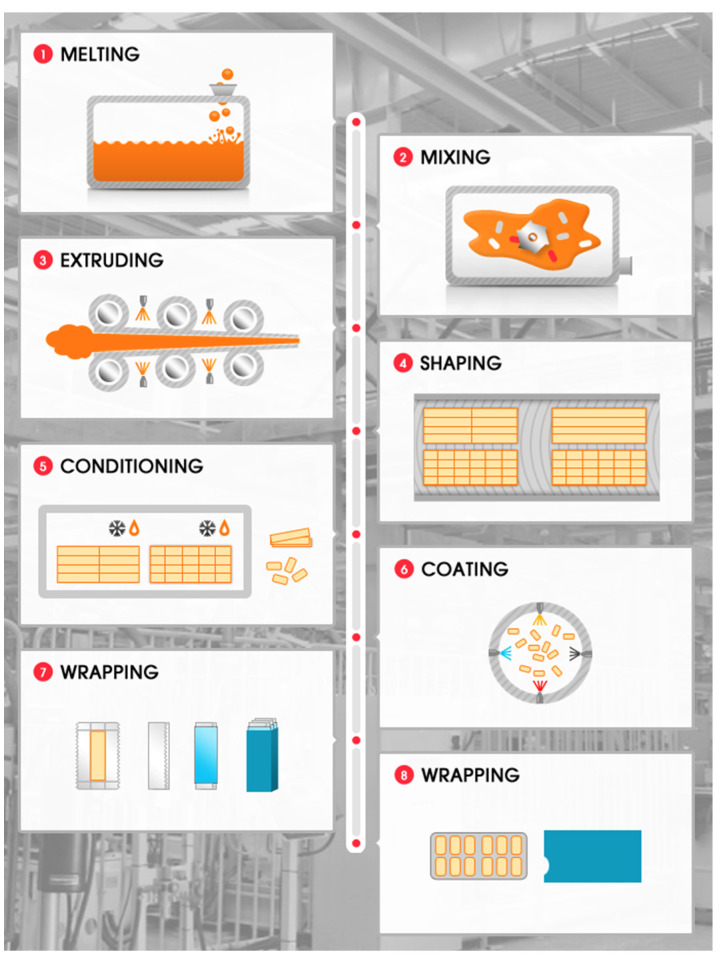
Schematic representation of conventional or fusion manufacturing method used for the preparation of medicated chewing gums. Steps include: (**1**) melting, (**2**) mixing, (**3**) extruding or rolling, (**4**) shaping or pattern selection, (**5**) conditioning (i.e., cooling and cutting), (**6**) coating, and (**7**,**8**) wrapping and packing. Reprinted with permission from [91].

The conventional or fusion method involves melting all the components of the gum base in a kettle with blades for mixing (Figure 3) [60]. The excipients are added in steps and are mixed at defined time points, whereas the drug is usually incorporated into the gum base before mixing it with other excipients to ensure its homogenous distribution [60]. The mixture is then passed through a series of rollers, forming a thin, wide ribbon. During this process, a light coating of finely powdered sugar or sugar substitutes can be added to prevent the gum from sticking and to improve its flavour. Subsequently, the gum is cooled for 48 h to allow it to set [60,90]. Finally, it is cut to the desired size and cooled under controlled temperature and humidity conditions [60]. Despite the simplicity of this approach, its main drawbacks lie in the stability issues associated with thermolabile drugs and the lack of precise form, shape or weight [89,90].

#### 4.2.2. Cooling, Grinding and Tableting

In this method, excipients of the gum base are cooled to a temperature at which the composition is sufficiently brittle and would remain brittle during the subsequent grinding step without adhering to the grinding apparatus [90]. Prior to the cooling step, some additives such as anti-caking (i.e., prevent agglomeration) and grinding agents (i.e., prevent the gum from sticking to the grinding apparatus) can be added to the mixture to facilitate cooling and grinding and to achieve the desired properties [89,90]. In general, the cooling temperature will differ depending on the composition of the chewing gum but is usually ≤−15 °C. Subsequently, the cooled mixture is crushed or ground to attain small fragments. Once the cooling agent is removed, the powder can then be mixed with the drug and the remaining excipients (e.g., binders, lubricants, flavouring agents, coating agents and sweeteners) in a blender. Finally, the mixture becomes ready for compression, which can be carried out using any conventional method, given that the humidity is strictly controlled [89,90]. The latter is considered the major limitation to this production pathway, and in fact, this method was developed to overcome the limitations of the fusion method [89].

#### 4.2.3. Direct Compression

The last manufacturing technique is the direct compression method [89,90,92]. It consists of free-flowing powders (e.g., Pharmagum) comprising a mixture of polyols, sugars, and gum base, which can be directly compacted using a conventional tableting machine, reducing manufacturing time and costs [89]. This manufacturing method accelerates the whole production process due to the mixture being directly compressible. However, the obtained chewing gums are generally harder and crumble during chewing, rendering them unpleasant to the patient [89].

Further information about the recent advances in medicated chewing gums preparation methods and mechanisms can be found in this review paper [92].

### 4.3. Chewable Lozenges

Depending on their texture and composition, lozenges can be categorised into hard lozenges, soft lozenges, chewable lozenges and compressed lozenges. Due to the focus of this review, only chewable lozenges will be discussed thereafter.

In chewable or caramel-based lozenges, drugs are incorporated into a caramel base (i.e., glycerinated gelatine base), and the dosage form should be chewed instead of being dissolved in the mouth, delivering the drug product down the GI tract for systemic absorption [93]. Typically, the formulation consists of glycerine, gelatine, and water [94]. These lozenges are often highly fruit flavoured and may have a slightly acidic taste to mask the acrid taste of glycerine [93]. The candy base is made up of a mixture of sugar and corn syrup in ratios of 50:50 to 75:25 [95]. Whipping agents (e.g., milk protein, egg albumin, gelatine, xanthan gum, starch, pectin, alginate, and carrageenan) are used in these types of lozenges to obtain the desired degree of soft chew [95]. The manufacturing process involves several steps [94,95]. First, the candy base is cooked at 95–125 °C and is transferred to a planetary/sigma blade mixer. The mass is then cooled, and the whipping agent is added below 105 °C. Subsequently, the drug is incorporated between 95–105 °C. The colourant is then dispersed in humectants and added below 85 °C, which is followed by the addition of the lubricant. Finally, the mixture is rolled into long strands of suitable thicknesses and thereafter cut into desired sizes. The formed lozenges must be cooled as quickly as possible to prevent loss of shape. To do so, they are usually cooled on a conveyor belt made of chains or canvas. Once collected, the properly sized lozenges must be stored in a climate-controlled room at 15–20 °C and relative humidity of 25–35%.

## 5. 3D Printing of Chewable Tablets: An Innovative Approach

Typically, medicines are manufactured in large batches with fixed doses through multi-step processes that are performed in centralised locations. Recently, with the introduction of new production technologies, the pharmaceutical industry has experienced a paradigm shift, causing treatments to move away from “one-size-fits-all” approaches and advance towards “precision medicine”. Precision or personalised medicine focuses on addressing the specific needs of patients and their medical condition, taking into account their genetic makeup and the inherent properties of the pharmaceutical product [96]. Thus, the overall goal is to improve the efficacy of the treatment whilst ensuring unwanted side effects are reduced.

In this new healthcare model, the end user’s needs and preferences are considered from the beginning of the formulation design stage to the point of administering the final product [97,98]. Personalised therapy has long been a remarkable goal in therapeutics but has not been adopted yet, mainly because of the lack of necessary tools and incentives, economic barriers as well as insufficient medical and pharmaceutical professionals willing [97]. As current production methods are wholly unsuitable for personalisation, this calls for the need for new manufacturing methods that are both simple and flexible, permitting the on-demand fabrication of medicines.

The 3D printing technology has been identified as a disruptive force in other fields, making it well suited for this application [14,99,100,101]. It is an additive manufacturing technology that enables the layer-by-layer fabrication of 3D objects based on digital 3D designs, created using a computer-aided design (CAD) software or obtained via 3D imaging techniques [11]. Although 3D printing is well-known in the automobile, aerospace and engineering fields, its use within the pharmaceutical space is somewhat new [102]. In fact, attention was drawn to it in 2015, following the FDA approval of the first 3D printed medicine (Spritam, levetiracetam) [103]. Since then, abundant research has been done on 3D-printed medicines and medical devices [99,104,105,106], with several attempts being made to launch 3D-printed drug products on the market. The main motivation behind the interest in this technology is its versatility and ability to customise doses, sizes, shapes and drug release profiles of small batches of medicines in a short time frame [107,108,109]. Thus far, its applications have extended to include personalised medicines, tissue engineering [110], controlled-release systems, as well as customised food products for specific needs [111,112,113]. Therefore, with this in mind and with the presence of suitable materials, 3D printing can be regarded as an ideal alternative method for producing personalised chewable tablets.

According to the American Society for Testing and Materials (ASTM) International, there are seven major 3D printing categories: binder jetting, vat polymerisation, powder bed fusion, material extrusion, material jetting, directed energy deposition, and sheet lamination [114]. Of these, material extrusion is the most widely used one and includes the fused deposition modelling (FDM) and semi-solid extrusion (SSE) technologies. In general, the material extrusion process involves selectively dispensing a material through an orifice with the aid of heat [114]. In FDM, filaments are melted through a heated nozzle at a specific temperature, after which the material is deposited on the build plate to form the layers [115,116,117]. While SSE operates in a similar fashion, syringes containing gels, pastes or waxes are used instead of filaments [118,119,120].

SSE is an affordable 3D printing technology that can offer many advantages in this field [118]. As an example, the preparation of its ink is generally considered easy and requires a few excipients. Due to the nature of the starting materials, SSE can employ lower printing temperatures compared to FDM, making it suitable for use with thermolabile drugs [121]. Additionally, the use of disposable and pre-filled syringes provides benefits for meeting the critical quality attributes demanded by regulatory agencies [118,122]. In particular, this enables the syringes to be prepared and filled as per GMP requirements at normal pharmaceutical production facilities. Furthermore, cross-contamination between different drugs or formulations can be avoided without the need for additional decontamination steps.

**Figure 4:**
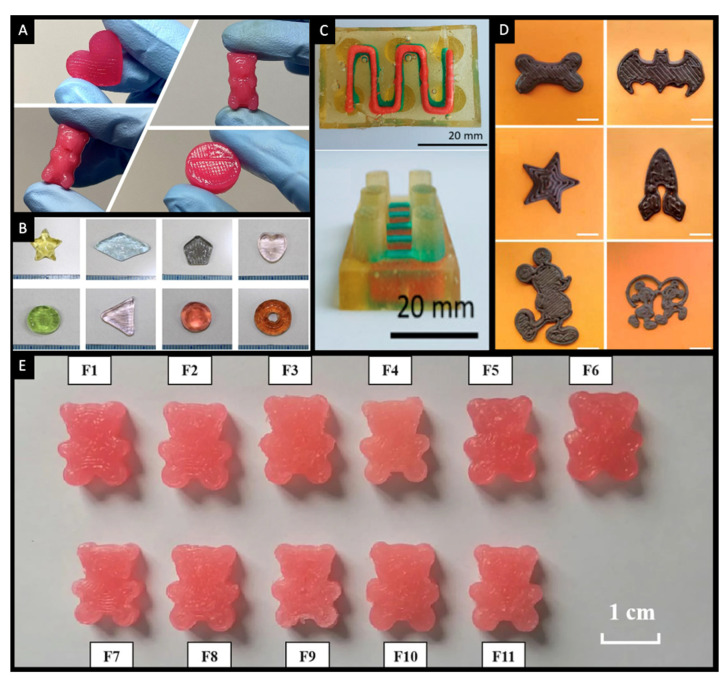
Images of various chewable 3D printed formulations fabricated using the SSE technology. (**A**) 3D-printed gummies in different shapes: heart, gummy bear and disk [126]. (**B**) 3D-printed jelly-like formulations in various shapes and colours [123]. (**C**) Lego-like gelatine-based dosage form containing paracetamol (blue) and ibuprofen (red) [127]. (**D**) 3D-printed chocolate-based dosage forms in various designs (scale bar: 20 mm) [131]. (**E**) Gummy bear-shaped chewable tablets made using 11 different formulations based on gelatine and carrageenan [134]. All images were reprinted with permission from their original sources.

**Figure 5:**
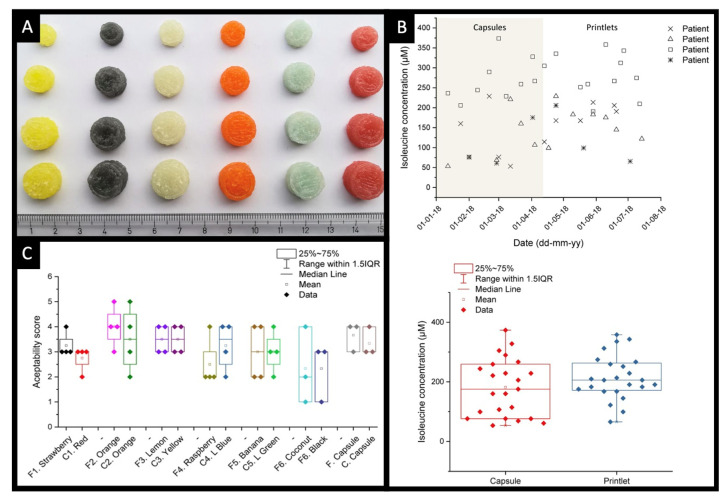
(**A**) Images of chewable isoleucine Printlets prepared in different sizes, flavours and colours. (**B**) (**top**) Isoleucine blood levels of the participants during the study, and (**bottom**) isoleucine blood levels and mean values for Printlets and capsules during the study. (**C**) Patient-reported outcome scores for the flavour and colour of the chewable Printlets and the capsule. F and C refer to the flavour and the colour of the formulations, respectively. Reprinted with permission from [132].

To date, the SSE technology has been successfully used for the preparation of a wide range of chewable formulations in different shapes, colours and textures (Figure 4) [123,124,125,126,127,128,129,130,131,132,133]. The most notable example is its use for the fabrication of isoleucine Printlets for children suffering from Maple Syrup Urine Disease, a rare metabolic disease characterised by the deficiency of the enzyme complex branched-chain alpha-keto acid dehydrogenase (Figure 5A) [132]. A clinical study involving the use of these Printlets has shown their ability to provide tighter control over the blood levels of isoleucine compared to treatment provided using conventional capsules (Figure 5B). Furthermore, children receiving the treatment and their caregivers have shown positive responses indicating their acceptability to the flavoured Printlets, with some flavours (e.g., orange) being more preferred over the other flavours (Figure 5C). These findings have shed light on the potential of the SSE technology as a novel pharmaceutical production method for manufacturing personalised oral dosage forms.

**Figure 6:**
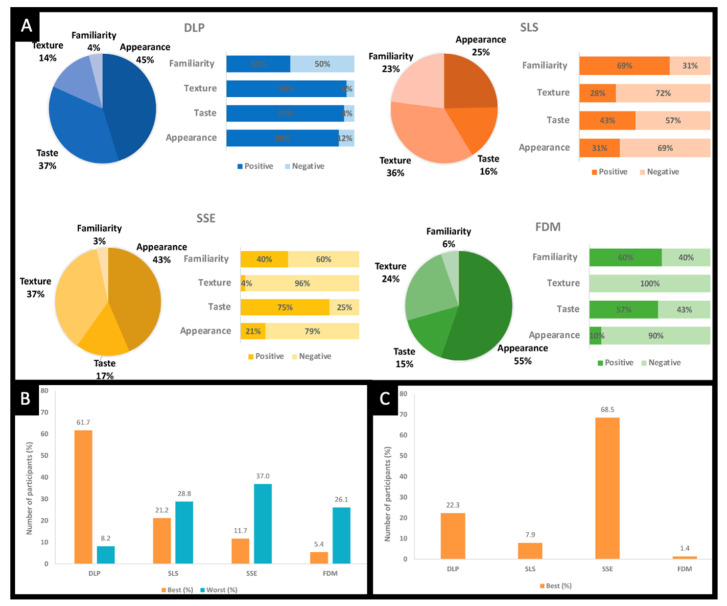
(**A**) Summary of visual description data for Printlets created using different 3D printing technologies based on four categories: appearance, perceived taste, texture and familiarity (DLP *n* = 244, SLS *n* = 170, SSE *n* = 125, FDM *n* = 92). Printlet visual preference results summary (**B**) before and (**C**) after the participants knew the SSE Printlets are chewable (*n* = 368). Reprinted with permission from [135].

A following study involved comparing children’s perceptions of Printlets made using different 3D printing technologies (i.e., FDM, digital light processing (DLP), selective laser sintering (SLS) and SSE) (Figure 6A) [135]. Despite the DLP Printlets being initially the preferred choice of the participants (aged 4–11 years) (Figure 6B), after being informed that SSE Printlets are chewable, the participants changed their minds, and 79% of them were in favour of the chewable Printlets (Figure 6C). In another acceptability study, it was shown that the shape, size and colour of Printlets could influence patients’ willingness to take them [136], thus highlighting the importance of selecting a dosage form that meets a patient’s particular preference to ensure his/her adherence to treatment.

**Figure 7:**
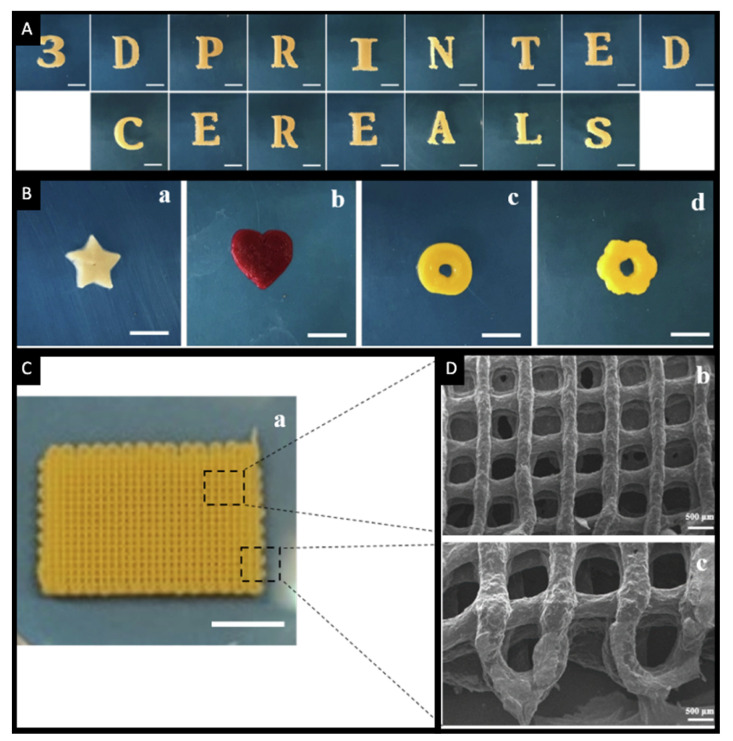
Images of SSE 3D printed cereal-based formulations in different shapes: (**A**) number and letters of the alphabet, (**B**) star (a), heart (b), torus (c) and flower (d), (**C**) a film (a). (**D**) Microscopic images (b,c) of the 3D printed film shown in (**C**). Red and yellow food colourings were used during cereal ink preparation. Scale bars for (**A**,**B**): 10 mm, (**C**): 5 mm (a) and (**D**): 500 μm (b,c). Reprinted with permission from [137].

The versatility of the 3D printing technology could be exploited to create multi-drug formulations termed PolyPrintlets. An example of such is the Lego-like chewable dosage forms fabricated using SSE (Figure 4C) [127]. The gelatine-based formulations contained a combination of paracetamol and ibuprofen and are aimed at simplifying administration by being dispensed as a single dosage form that provides a synergistic therapeutic effect. In another approach, it has been shown that it is also possible to fabricate chocolate-based dosage forms for paediatric applications (Figure 4D) [131]. The formulations were loaded with either paracetamol or ibuprofen, wherein the inherent drug properties governed its release behaviour. More recently, cereal-based 3D printed dosage forms have been suggested for paediatric use [137]. The concept involved concealing the drugs, namely ibuprofen and paracetamol, in a common breakfast ingredient, cereals. Herein, the crushed cereal was used as the ink for SSE 3D printing of oral formulations in different shapes (e.g., various letters, star, heart, torus and flower shapes) (Figure 7). These formulations are aimed at improving adherence to treatment in paediatric patients during their hospital stay.

Owing to the digitised nature of the technology, it is forecast that in the future, 3D printing could be seamlessly integrated with other digital technologies, including artificial intelligence [138,139,140], biosensors [141,142] and robots [143,144], streamlining a new era of digital healthcare [145,146]. With the aid of these technologies, the personalisation of medicines can be facilitated by expediting the process and enabling execution in remote locations, including patients’ homes. In fact, research in this area has already begun with the introduction of smartphone-enabled 3D printing [147]. This recently developed technology involves the use of a smartphone’s screen to initiate the 3D printing of medicines inside a compact, portable 3D printing system. Whilst the concept is still in its infancy, more advancements are expected in the near future, fast-forwarding the implementation of 3D printing in clinical practice.

## 6. Excipients for Chewable Medicines

Typically, when formulating a medicine, the choice of excipients will depend on a number of factors, such as the type of dosage form, the production method, the API properties and the intended drug release profile [148]. Table 2 provides a summary of commercialised chewable medications and the excipients used in their formulation. It is essential to note that the key excipients listed in Table 1 (Section 4.1), which are used for manufacturing chewable tablets using conventional methods, can also be used for the preparation of 3D printed chewable formulations. To date, the use of 3D printing has been focused on jelly-like chewable formulations. Thus, the use of gelling agents has become common [149]. This is due to their ability to modify the formulation’s rheological properties, including its viscosity and texture [150].

The gelation process involves the entanglement of randomly dispersed polymer chains in such a way that they form 3D networks that contain solvents in their interstices. The main mechanisms of physical entanglement include ionotropic (i.e., crosslinking with ions), cold-set or heat-set gelation. The entangled regions, known as “junction zones”, may be formed by two or more polymer chains, wherein the resulting number and strength of junctions are affected by several factors (e.g., the concentration of the gelling agent, temperature, pH and presence or absence of ions) [151].

The most commonly used gelling agents include gelatine, starch, pectin, carrageenan and alginate. Agar, cellulose derivatives (e.g., carboxymethyl cellulose, hydroxypropyl cellulose, hydroxypropyl methyl cellulose, ethyl cellulose), chitosan, hyaluronic acid, collagen and gellan gum have also been tested [150,152]. Whilst some gelling agents have the ability to spontaneously form gels, a few of them (e.g., xanthan and guar gum) must be coalesced together to improve their viscosity or induce the gelation process. It must be noted that the choice of gelling agent is critical for 3D printing. This is because modifying the ink’s viscoelasticity will impact the printing resolution and precision and, consequently, the final geometry of the dosage form.

## 7. Veterinary Applications

Veterinary pharmaceuticals play an important role in the preservation and restoration of animal health [173]. In the veterinary field, animal-appropriate medicines, which are available in a wide range of dosages, are also required to meet animals’ needs. Species differences affecting the design and performance of veterinary dosage forms include pharmacokinetic differences, feeding habits, environmental factors, age and management practices [174]. Generally, the medicine’s dose is adjusted based on the weight of the animal [173]. Therefore, it is common for a drug to be marketed with several strengths. This is best exemplified with fluralaner, clindamycin hydrochloride and mavacoxib (Table 3). Alternatively, it is ordinary practice for vets and pet owners to split marketed tablets into two or four pieces to meet an animal’s requirements (e.g., dose or swallowing abilities). Like humans, animals have preferences that affect their compliance and willingness to take a medicine [174]. Thus, when a veterinary medicine is developed, animals’ preferences are an important aspect to consider. For instance, dogs prefer animal-based proteins (e.g., chicken, pork and beef), whilst horses like fruit flavours (e.g., apple). As such, the Simparica Trio product contains pork liver powder, hydrolysed vegetable protein, sugars, and gelatine to address dog-specific sensory requirements.

Historically, oral dosage forms and parenteral formulations have been the primary dosage forms used for animal care [174]. Nowadays, with the advancement in pharmaceutical production, several more convenient oral dosage forms (e.g., palatable tablets) have been launched [174]. Indeed, chewable tablets have found applications in veterinary medicine for administration to domestic animals, especially cats [175] and dogs [176]. In fact, chewable tablets play a more essential role in veterinary pharmaceuticals than human ones. A summary of chewable formulations available on the market for animal use can be found in Table 3. As a matter of fact, the number of commercialised chewable formulations for veterinary use exceeds those for humans. A reason for this may be their easier administration due to the animal’s willingness to ingest the medicine.

**Figure 8:**
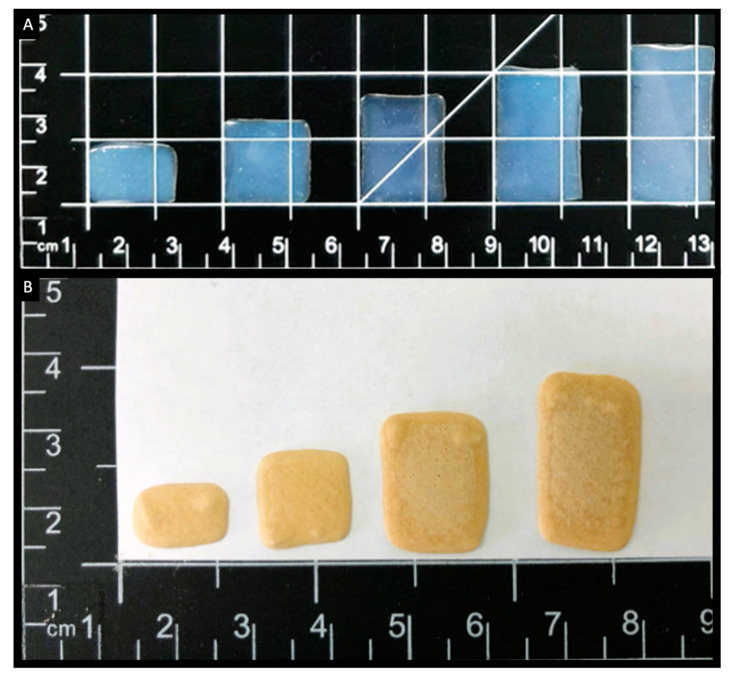
Images of SSE 3D printed (**A**) prednisolone-loaded films [220] and (**B**) theophylline chewable tablets [221], in different sizes. All images were reprinted with permission from their original sources.

The benefits of 3D-printed medicines are not only limited to humans but can also extend to include veterinary applications. In this regard, 3D printing has been used for the production of animal prosthetics and implants [217,218,219] as well as veterinary dosage forms. Representative examples include orodispersible films containing prednisolone for the treatment of inflammatory diseases in cats and dogs (Figure 8A) [220], chewable tablets (or ChewTs) containing theophylline for the treatment of asthma (Figure 8B) [221] or gabapentin for the treatment of neuropathic pain or prevention of seizures [222], both for use in cats and dogs. Dosage forms with precise doses and palatability could be 3D printed, especially using SSE technology, in the veterinary clinic or at the owner’s home to ensure their suitability for the pet [11,220]. Further examples on 3D printing for animal use can be found in previous reviews [223,224].

## 8. Considerations and Requirements of Chewable Tablets—A Regulatory Aspect

As mentioned in previous sections, a chewable tablet must ideally be [41]: easy to chew, palatable, have an appropriate size and shape, and disintegrate readily. In this section, the main recommendations and assays that should be carried out to ensure the quality of chewable tablets are discussed [41]. It must be noted that these methods are applicable for both chewable and swallowable tablets due to the similarities between both.

### 8.1. Mechanical Properties

According to the USP, mechanical tests that are used to indirectly assess chewability include hardness (also known as “breaking force”), tensile strength, and the recently developed chewing difficulty index. Hardness refers to the force needed to break a tablet in a specific plane and may be expressed in a variety of units (e.g., kilopond (kp), kilogram-force (kgf), Newton (N), and Strong–Cobb Units (scu)) [225]. The tablet is placed between two platens across its diameter, wherein one of the platens moves and applies force until the tablet fractures. Typically, for chewable tablets, hardness values below 12 kp are recommended by the FDA. However, higher values may be allowed if the tablet’s hardness reduces after exposure to saliva [41]. Hardness plays an important role because chewable tablets with high mechanical strength have a high risk of breaking teeth, dentures, or mandibular joints. Ideally, chewable tablets should be hard enough to resist the rigors of manufacturing, packaging, shipping, and distribution but should not cause harm to the patient during administration [41]. Despite the extensive use of hardness for the determination of a tablet strength, variations related to inaccuracies in the instrumental scales of different apparatuses, the load application method and the size or geometry of the tablet have been reported [226,227,228,229].

**Figure 9:**
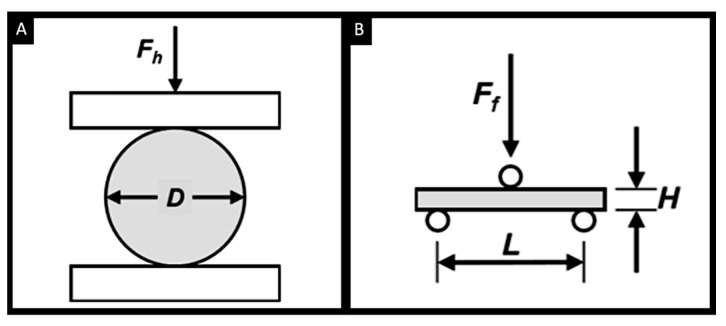
Graphical illustration of the two methods used to calculate the tensile strength: (**A**) diametrical tensile strength test and (**B**) flexure tensile strength test. Reprinted with permission from [232].

Two methods are used for measuring a tablet’s tensile strength: (a) diametral compression or the diametrical tensile strength test (Figure 9A) [230], and (b) flexural bending or the flexure tensile strength test (Figure 9B) [231].

The diametral tensile strength (σ_h_) is calculated using:

σ_h_ = 2·F_h_/π·D·H (1)

where F_h_ is the load or force needed to break a tablet (also known as hardness or breaking force), D is the diameter of the tablet, and H is its thickness.

The flexure tensile strength (σ_f_) can be calculated using:

σ_f_ = 3·F_f_·L/2·D·H^2^ (2)

where F_f_ is the force needed to break a tablet under flexural or bending stress, and L is the constant distance between the two lower supports.

Although the tensile strength values calculated by the two methods are different, they are proportional to one another [229,231]. The relationship between the two tensile values can be deduced from the following equation:

σ_f_ = k·σ_h_ (3)

where k is the constant of proportionality. Substituting Equations (1) and (2) in Equation (3) results in the following:

F_f_ (3·π·L/4·k) = F_h_·H (4)

Since 3πL is an experimental constant and k is the constant of proportionality, the chewing difficulty index (CDI) has been proposed as a measure of the ease or difficulty of chewing a chewable tablet and is defined as [233]:

CDI = F_h_·H (5)

Although the tensile strength provides a more fundamental measure of a tablet’s strength due to its independence of the size and measurement method, it is only limited to cylindrical tablets. Thus, CDI values can be used as an alternative when it is not possible to measure the tensile strength [233,234].

### 8.2. Disintegration and Dissolution

Chewing a dosage form helps reduce its size, enabling it to be swallowed more easily, especially in the case of large tablets. However, in some cases, patients may choose to swallow an entire chewable tablet without mastication or without chewing the dosage form enough before swallowing it, posing a risk for potential GI obstructions. This can be avoided by formulating chewable tablets to have a rapid disintegration time. The latter refers to the time needed for a tablet to break up into small pieces. In vitro disintegration testing should be performed in a suitable medium [41], using established and validated disintegration equipment (e.g., basket-rack assembly or disks [41,235]). Ideally, the tests should be carried out using intact tablets to predict their behaviour if swallowed whole. Although the FDA recommends a disintegration time short enough to prevent GI obstruction, no specific values have been described [41]. It is important for manufacturers to emphasise on the product label that these tablets must be chewed before swallowing, avoiding any free interpretations by the end-users and ensuring patient safety. For dissolution testing, the FDA also recommends carrying out the experiments using intact tablets [41] whilst employing validated methods [e.g., the basket method (USP apparatus I) and the paddle method (USP apparatus II) [236].

It is advisable that chewable tablets meet the same disintegration and dissolution specifications as immediate-release tablets [41]. It should be noted, however, that these testing conditions do not entirely mimic realistic conditions of chewable tablets. Instead, more research is needed to develop new validated methods that are specific to these dosage forms. In particular, the methods must replicate the chewing of the dosage forms before performing in vitro dissolution tests. However, there are several aspects to take into consideration; for one, it could be difficult to validate such methods that simulate and mimic the chewing patterns, especially since they vary based on patient populations. Moreover, it is challenging to assess whether the proposed methods are equivalent or superior to the existing approaches or not.

## 9. Conclusions

Chewable tablets are dosage forms suitable for use in certain patient populations, especially paediatrics, geriatrics and those who suffer from dysphagia, complying with their individual requirements. Despite the advantages that chewable formulations offer, the current methods used for their production are inherently time-consuming and inflexible, making it difficult to optimise the dosage form characteristics based on the individual needs and preferences of patients, both of which affect their adherence to the therapeutic plan. In addition, it can be noted that chewable dosage forms are widely used in routine clinical practice, both for humans and animals, as shown in Table 2 and Table 3, respectively. However, there is still a need for new approaches capable of addressing the limitations of conventional manufacturing methods.

Recently, 3D printing, in particular the SSE technology, has gained attention as a novel fabrication method for the production of chewable medicines. The implementation of this disrupting approach is set to revolutionise the way dosage forms are fabricated in the near future. This technology can create palatable dosage forms with personalised doses, shapes, colours and textures in a simple and fast process, using the same excipients as conventional chewable tablets and, therefore, making it superior to manufacturing methods currently in use. This statement is reflected in many of the articles cited in this review, in which the semi-solid extrusion technology was successfully used to prepare bespoke chewable formulations.

Indeed, this innovative concept has already been tested in a clinical trial performed in a hospital setting with children, wherein the positive findings are a testament to SSE technology’s great potential. More recently, further studies were carried out in patients, wherein the application of chewable formulations can be further understood; one such included an acceptability study related to children’s perceptions of Printlets (3D printed oral dosage forms) made using different 3D printing technologies. Although SSE Printlets were not originally the participants’ top choice, after being informed that SSE Printlets were chewable, the majority of participants shifted their preference in favour of the chewable Printlets.

The benefits of 3D printing are not only limited to human healthcare but also extend to veterinary medicine, where both vets and pet owners could exploit it to create customisable formulations in a fast and simple manner, avoiding dosing errors or the animals’ rejection of unpalatable medicines. Although progress has been made in the use of 3D printing for the preparation of chewable formulations, a myriad of research is yet to be done with regards to the selection of appropriate starting materials (especially gelling agents) and the characterisation of the rheological properties (mainly the viscosity) of formulations suitable for 3D printing.

From a regulatory perspective, chewable tablets are treated similar to conventional tablets, with disintegration and dissolution assays conducted on whole tablets in the same manner as swallowable tablets. However, in reality, the in vivo performance of these formulations differs from that observed during in vitro tests due to the absence of a step that mimics the chewing process. Regarding their mechanical properties, the chewing difficulty index (CDI) has been recently proposed as a quantitative measurement of the ease or difficulty of chewing a chewable tablet and is increasingly being used by researchers. Nevertheless, there is still no significant progress in developing methods that can evaluate chewable formulations following masticating. Thus, researchers should be encouraged to develop new validated methods to evaluate chewable dosage forms under realistic conditions.

## Figures and Tables

**Table 1:** Summary of the main excipients used in chewable tablets with their functions and examples.

Excipient	Function(s)	Example(s)
Sweeteners	Mask unpleasant taste, microbial stability	Polyols (mannitol, sorbitol, xylitol), sugars (dextrose, lactose, sucrose, saccharine, sucralose), aspartame
Flavouring agents	Mask unpleasant taste	Fruit-based agents (mint, strawberry)
Colourants	Enhance aesthetic appeal, identification of the product, mask non-uniform colour, match the flavour	Powder-based colourants
Diluents	Increase the bulk volume	Polyols
pH regulators	Acidity modifiers	Citric acid, malic acid
Gelling agents	Initiate gelation	Gelatine, cellulose derivatives, starch, pectin, carrageenan, alginate, chitosan, hyaluronic acid, collagen and gellan gum

**Table 2:** Examples of marketed chewable drug products for adult and paediatric use.

Active Pharmaceutical Ingredient(s)	Commercial Name	Dose(s)	Excipients	Indication(s)	Target Population(s)	Ref.
Montelukast sodium	Singulair paediatric chewable tablets	4 and 5 mg	Mannitol, microcrystalline cellulose, sodium croscarmellose, aspartame, magnesium stearate, hyprolose, cherry flavour	Chronic asthma, prevention of exercise-induced bronchoconstriction	Children (2–5 years old)	[153]
Lamotrigine	Lamictal	2, 5, 25 and 100 mg	Calcium carbonate, hydroxypropyl cellulose, sodium starch glycolate (Type A), povidone K30, saccharin sodium, magnesium stearate	Epilepsy, bipolar disorder	Adults, adolescents and children	[154]
Calcium carbonate/cholecalciferol	Calcichew-D3 chewable tablets	1 g/800 IU, 500 mg/200 IU, and 500 mg/400 IU	Hydrated lactose, aspartame, sodium croscarmellose, maltodextrin	Vitamin D and calcium deficiency	Adults	[155]
Calcium carbonate	Remegel	800 mg	Glucose syrup, sucrose, glycerol, hydrolysed milk protein, gelatine, sorbitol	Relief of acid indigestion and heartburn and associated stomach upsets (dyspepsia)	Adults and over 12 years old	[156]
Calcium carbonate	Children chewable antacid	400 mg	Sucrose, mannitol	Antacid	Children	[157]
Sodium alginate/potassium bicarbonate	Gaviscon advance mint chewable tablets	500/100 mg	Sodium, potassium, mannitol, macrogol 20,000, magnesium stearate, aspartame, acesulfame potassium, copovidone	Treatment of symptoms resulting from acid, bile and pepsin reflux into the oesophagus	Adults and children	[158]
Calcium carbonate/sodium bicarbonate/light magnesium carbonate	Bisodol original indigestion relief tablets	522/64/68 mg	Saccharin soluble, maize, starch, sugar, calcium stearate	Relief of the symptoms of gastric hyperacidity, including indigestion, heartburn, dyspepsia and flatulence	Adults and children over 12 years old	[159]
Lanthanum carbonate hydrate	Fosrenol chewable tablets	1000, 500 and 750 mg	Dextrates, anhydrous colloidal silica, magnesium stearate	Hyperphosphatemia in chronic renal failure	Adults	[160]
Magnesium glycerophosphate	Neomag	97 mg	Maize starch, microcrystalline cellulose, talc, aspartame, magnesium stearate, anhydrous colloidal silica, povidone	Magnesium supplements for the treatment of patients with chronic magnesium loss or hypomagnesaemia and hypomagnesaemia due to the concomitant administration of loop and thiazide diuretics or other drugs	Adults and children over 4 years old	[161]
Phenytoin	Epanutin Infatabs	50 mg	Confectioner′s sugar, saccharin sodium, spearmint flavour, magnesium stearate, talc, quinoline yellow (E104), sunset yellow FCF (E110)	Control of seizures and prevention and treatment of seizures occurring during or following neurosurgery and/or severe head injury	Adults and children	[162]
Acetaminophen	Children chewable acetaminophen	160 mg	Mannitol	Analgesic, antipyretic	Children	[163]
Aspirin	Bayer chewable aspirin	81 mg	Sucralose, maltodextrin, dextrose monohydrate, starch	Analgesic	Children	[164,165]
Raltegravir	Isentress	25 and 100 mg	Hydroxypropyl cellulose, sucralose, fructose, aspartame, sucrose, sorbitol, magnesium stearate	Treatment of HIV-1 infection	Adults and children	[166,167]
Loratadine	Children′s loratadine chewable tablets USP	5 mg	Aspartame, colloidal silicon dioxide, magnesium stearate, mannitol, microcrystalline cellulose, sodium starch glycolate	Relieve symptoms related to hay fever or other upper respiratory allergies	Children	[168]
Atorvastatin calcium trihydrate	Lipitor chewable tablets	10 and 20 mg	Aspartame	Hypercholesterolemia and prevention of cardiovascular disease	Adults, adolescents, children aged 10 years or older	[169]
Metoclopramide/dimethicone	Aeroflat chewable tablet	5/77.5 mg	Silicated microcrystalline cellulose, acesulfame K	Prevention and treatment of nausea and vomiting Symptomatic relief of aerophagia and meteorism	Adults	[170]
Sildenafil citrate	Nipatra chewable tablets	25, 50 and 100 mg	Polacrilin potassium, anhydrous colloidal silica, lactose monohydrate, povidone K-30, aspartame, sodium croscarmellose, magnesium stearate, sodium hydroxide or hydrochloric acid	Erectile dysfunction	Adults	[171]
Sucroferric oxyhydroxide	Velphoro chewable tablets	500 mg	Neohesperidin-dihydrochalcone, magnesium stearate, anhydrous colloidal silica	Control of serum phosphorus levels in chronic kidney disease	Adults and children	[172]

**Table 3:** Examples of marketed chewable drug products for veterinary use.

Active Pharmaceutical Ingredient(s)	Commercial Name(s)	Dose(s)	Excipients	Indication(s)	Animal(s)	Ref
Milbemycin oxime/Praziquantel	Aderexa and Interceptor plus	12.5/125 and 2.5/25 mg	Microcrystalline cellulose, lactose monohydrate, povidone, sodium croscarmellose, anhydrous colloidal silica, meat flavour, yeast powder, magnesium stearate	Treatment of mixed infections by adult cestodes and nematodes	Dogs	[177,178]
Amlodipine	Amlodip	1.25 mg	Artificial chicken flavour, malted yeast, microcrystalline cellulose, mannitol sodium croscarmellose, magnesium stearate, anhydrous colloidal silica	Treatment of systemic hypertension	Cats	[179,180]
Oclacitinib maleate	Apoquel	3.6, 5.4 and 16 mg	Pork liver powder, crospovidone (Type A), sodium starch glycolate (Type A), glycerol monostearate 40–55 (Type II), macrogol 3350, glycerol sodium chloride, xanthan gum, brewer’s dried yeast, anhydrous colloidal silica, magnesium stearate	Treatment clinical manifestations of allergic and atopic dermatitis	Dogs	[181]
Fluralaner	Bravecto	112.5, 250, 500, 1000 and 1400 mg	Pork liver flavour, sucrose, maize starch, sodium lauryl sulphate, disodium embonate monohydrate, magnesium stearate, aspartame 6, glycerol, soya-bean oil, macrogol 3350	Treatment of tick and flea infestations	Dogs	[182,183]
Benazepril Hydrochloride/spironolactone	Cardalis	2.5/20, 5/40 and 10/80 mg	Lactose monohydrate, microcrystalline cellulose, povidone K30 8, artificial beef flavour, compressible sugar, crospovidone, magnesium stearate	Treatment of congestive heart failure caused by chronic degenerative valvular disease	Dogs	[184]
Carprofen	Carprodyl Quadri	120 mg	Pig liver flavour, yeast, sodium croscarmellose, copovidone, magnesium stearate, anhydrous colloidal silica, microcrystalline cellulose, lactose monohydrate	Anti-inflammatory and analgesic in musculoskeletal disorders and degenerative joint disease	Dogs	[185,186]
Cimicoxib	Cimalgex	8, 30 and 80 mg	Lactose monohydrate, povidone K25, crospovidone, s odium lauryl sulphate, macrogol 400, sodium stearyl fumarate, pork liver powder	Analgesic and anti-inflammatory in osteoarthritis, perioperative pain due to orthopaedic or soft tissue surgery	Dogs	[187]
Amoxicilin/Clavulanic acid	Cladaxxa	40/10, 200/50 and 400/100 mg	Microcrystalline cellulose, magnesium stearate, anhydrous colloidal silica, sodium starch glycollate (type A), dried autolysed yeast, erythrosine aluminium lake E127	Treatment of infections caused by susceptible bacteria in skin, soft tissue, dental tissue, urine tract, respiratory tract and gut	Cats and dogs	[188]
Clindamycin hydrochloride	Zodon (dogs only)	55, 88, 150, 220, 264 and 440 mg	Zodon: chicken flavour, yeast extract, sodium croscarmellose, copovidone, magnesium stearate, anhydrous colloidal silica, microcrystalline cellulose, lactose monohydrate	Cats: treatment of infected wounds and abscesses and oral cavity infections, including periodontal disease, caused by susceptible bacteria	Cats (only 55 mg) and dogs	[189,190]
	Clindabactin (cats and dogs)	55, 220 and 440 mg	Clindabactin: croscarmellose sodium, pregelatinised maize starch, microcrystalline cellulose, hydrated colloidal silica, yeast (dried), chicken flavour, magnesium stearate	Dogs: treatment of infected wounds and abscesses, oral cavity infections (including periodontal disease), superficial pyoderma and osteomyelitis caused by susceptible bacteria		
Spinosad A/D 85:15	Comfortis	90, 140, 180, 270, 425, 665, 1040 and 1620 mg	Microcrystalline cellulose, artificial beef flavour, hydroxypropyl cellulose, colloidal silicon, anhydrous, croscarmellose sodium, magnesium stearate	Treatment and prevention of flea infestations	Cats (except 665, 1040 and 1620 mg) and dogs	[191]
Lotinaler	Credelio	12, 48, 56, 112, 225, 450 and 900 mg	Cellulose powdered, lactose monohydrate, Silicified microcrystalline cellulose, dry meat flavour (not in cats), crospovidone, povidone K30, sodium lauryl sulphate, anhydrous colloidal silica, magnesium stearate	Treatment of flea and tick infestations	Cats (only 12 and 48 mg) and Dogs (only doses ≥ 56 mg)	[192]
Lotilaner/Milbemycin Oxime (A3 and A4)	Credelio Plus	56.25/2.11, 112.5/4.22, 225/8.44, 450/16.88 and 900/33.75 mg	Cellulose powdered, lactose monohydrate, silicified microcrystalline cellulose, dry meat flavour, crospovidone, povidone K30, sodium lauryl sulphate, silica colloidal anhydrous, magnesium stearate	Treatment of mixed infestations/infections of ticks, fleas, gastrointestinal nematodes, heartworm and/or lungworm	Dogs	[193]
Dexamethasone	Dexacortone	0.5 and 2 mg	Lactose monohydrate, potato starch, povidone K30, magnesium stearate, chicken flavour, yeast (dried)	Symptomatic treatment or as adjunct treatment of inflammatory and allergic conditions	Cats and Dogs	[194]
Marbofloxacin	Efex	10, 40 and 100 mg	Lactose monohydrate, copovidone, silica colloidal anhydrous, croscarmellose sodium, hydrogenated castor oil, pig liver powder, malted yeast, microcrystalline cellulose	Cats: skin and soft tissue infections (wounds, abscesses, phlegmons) and upper respiratory tract infections caused by susceptible strains Dogs: skin and soft tissue infections (skinfold pyoderma, impetigo, folliculitis, furunculosis, cellulitis), UTI associated or not with prostatitis or epididymitis and respiratory tract infections caused by susceptible strains	Cats and Dogs	[195]
Ivermectin/Praziquantel	Equimax	150/20 mg	Povidone, crospovidone, microcrystalline cellulose, cider applemarc (pressed apple pulp), glucose, pregelatinized liquid starch, compressible sugar, magnesium stearate	Treatment of mixed cestode, nematode and arthropod infestations	Horses	[196]
Ivermectin/Pyrantel pamoate	Cardotek 30 plus	68 µg/163 mg, 136 µg/326 mg and 272 µg/652 mg	Polyoxyl 40, hydrogenated castor oil, distilled monoglycerides, ground corn cob, formulated antioxidant, tallow, lean beef, refined soy protein, purified water, dextrose, propylene glycol, sodium chloride, ethoxyquin, potassium sorbate, delta gluconolactone	Prevention of canine heartworm and treatment of infestations of nematodes (ascarids and hookworms)	Dogs	[197]
Ivermectin	Eraquell	20 mg	Povidone, crospovidone, microcrystalline cellulose, cider applemarc (pressed apple pulp), glucose, pregelatinised liquid starch, compressible sugar, magnesium stearate	Treatment of nematode and arthropod infestations	Horses	[198]
Firocoxib	Equioxx (horses) and Firodyl	57, 227 and 250 mg	Equioxx: lactose monohydrate, microcrystalline cellulose, chartor hickory smoke flavour, hydroxypropyl cellulose, sodium croscarmellose, magnesium stearate, caramel (E150d), anhydrous colloidal silica, yellow iron oxide (E172), red iron oxide (E172) Firodyl: hydroxypropyl cellulose, sodium croscarmellose, microcrystalline cellulose, anhydrous colloidal silica, lactose monohydrate, magnesium stearate, yeast, chicken flavour	Horses: Alleviation of pain and inflammation associated with osteoarthritis and reduction of associated lameness Dogs: Relief of pain and inflammation associated with osteoarthritis or For post-operative pain and inflammation associated with soft-tissue, orthopaedic and dental surgery	Horses (only 57 mg) and Dogs	[199,200]
Afoxolaner	Frontpro and NexGard	11, 28, 68 and 136 mg	Maize starch, soy protein fines, braised beef flavouring, povidone (E1201), macrogol 400, macrogol 4000, macrogol 15 hydroxystearate, glycerol (E422), medium-chain triglycerides.	Treatment of flea and tick infestations, demodicosis and sarcoptic mange	Dogs	[201,202]
Meloxicam	Inflacam	1 and 2.5 mg	Lactose monohydrate, silicified microcrystalline cellulose, sodium acid citrate, crospovidone, talc, pork flavour, magnesium stearate	Alleviation of inflammation and pain in chronic musculoskeletal disorders	Dogs	[203]
Torasemide	Isemid	1, 2 and 4 mg	Lactose monohydrate, microcrystalline cellulose, povidone (K30), pork liver powder flavour, compressible sugar, crospovidone (type B), magnesium stearate	Treatment of clinical signs related to congestive heart failure in dogs, including pulmonary oedema	Dogs	[204]
Furosemide	Libeo	10 and 40 mg	Chicken flavour, yeast extract, maltodextrin, magnesium stearate, anhydrous colloidal silica, microcrystalline cellulose, sodium croscarmellose, lactose monohydrate	Treatment of ascites and oedema, particularly associated with cardiac insufficiency	Dogs	[205]
Sarolaner	MiPet Easecto	5, 10, 20, 40, 80 and 120 mg	Hypromellose acetate succinate (medium grade), lactose monohydrate, sodium starch glycolate, anhydrous colloidal silica, magnesium stearate, maize starch, confectioner’s sugar, glucose liquid (81.5% solids), spray-dried pork liver powder, hydrolysed vegetable protein, gelatine type A, wheat germ, calcium hydrogen phosphate anhydrous	Treatment of tick, flea, sarcoptic mange and ear mite infestations	Dogs	[206]
Afoxolaner/Milbemycin Oxime (A3 and A4)	Nexgard Spectra	9/2, 19/4, 38/8, 75/15 and 150/30 mg	Maize starch, soy protein fines, braised beef flavouring, povidone (E1201), macrogol 400, macrogol 4000, macrogol 15 hydroxystearate, glycerol (E422), triglycerides medium-chain, citric acid monohydrate (E330), butylhydroxytoluene (E321)	Treatment of flea and tick infestations, concurrent prevention of heartworm disease, angiostrongylosis, thelaziosis and/or treatment of GI nematode infestations. Treatment of demodicosis and sarcoptic mange. Prevention of heartworm disease and angiostrongylosis	Dogs	[207]
Pimobendan	Pimotab	1.25, 5, 10 and 15 mg	Citric acid anhydrous, povidone K25, lactose monohydrate, microcrystalline cellulose, sodium croscarmellose, chicken flavour, yeast, hydrated colloidal silica, magnesium stearate	Treatment of congestive heart failure originating from dilated cardiomyopathy or valvular insufficiency	Dogs	[208]
Phenylpropanolamine hydrochloride	Proin	15 and 50 mg	Calcium hydrogen phosphate dehydrate, anhydrous colloidal silica, sorbitol, stearic acid, whey, powdered soy protein concentrate, chicken liver powder, dry liver flavour, dry garlic flavour, garlic powder, brewer’s yeast, dark brown lake LB506	Management of urinary incontinence associated with urethral sphincter incompetence in the bitch, particularly that associated with ovariohysterectomy	Dogs	[209]
Moxidectin/Pyrantel embonate/Sarolaner	Simparica Trio	0.06/12.5/3, 0.12/25/6, 0.24/50/12, 0.48/100/24, 0.96/200/48 and 1.44/300/72 mg	Hypromellose, lactose monohydrate, sodium starch glycolate (type A), meglumine, butylhydroxytoluene (E321), pigment blend 018 (E110, E129, E132), hydroxypropyl cellulose, anhydrous colloidal silica, magnesium stearate, maize starch, confectioner’s sugar, glucose liquid, pork liver powder, hydrolysed vegetable protein, gelatine, wheat germ, calcium hydrogen phosphate anhydrous	Treatment of mixed external and internal parasitic infestations (fleas, ticks and nematodes infestations)	Dogs	[210,211,212]
Spiramycin/Metronidazole	Spizobactin	750,000 IU/125 mg, 1,500,000 IU/250 mg and 3,000,000 IU/500 mg	Pregelatinised starch, microcrystalline cellulose, lactose monohydrate, hydroxypropyl cellulose, yeast, chicken flavour, anhydrous colloidal silica, magnesium stearate	Adjunct treatment of mechanical or surgical periodontal therapy in the treatment of multi-bacterial infections of periodontal and related (peri)oral conditions	Dogs	[213]
Tramadol hydrochloride	Tralieve	20 and 80 mg	Microcrystalline cellulose, lactose monohydrate, sodium starch glycolate (type A), magnesium stearate, hydrated colloidal silica, chicken flavour, yeast	Reduction of acute and chronic mild soft tissue and musculoskeletal pain	Dogs	[214]
Mavacoxib	Trocoxil	6, 20, 30, 75 and 95 mg	Sucrose, silicified microcrystalline cellulose, artificial powdered beef flavour, sodium croscarmellose, sodium lauryl sulphate, magnesium stearate	Treatment of pain and inflammation associated with degenerative joint disease when continuous treatment exceeds one month	Dogs	[215]
Febantel/Praziquantel/Pyrantel	Veloxa and Veloxa XL	150/50/50 and 525/175/175 mg	Cetyl palmitate, pregelatinised starch, sodium starch glycolate (type A), anhydrous colloidal silica, magnesium stearate, artificial beef flavour	Anthelmintic for treatment of mixed infections by roundworms and tapeworms in dogs and puppies	Dogs (Veloxa XL over 17.5 kg)	[216]

## Data Availability

Not applicable.

## Supplementary Materials

The following supporting information can be downloaded at: https://www.mdpi.com/article/10.3390/pharmaceutics14081732/s1, File S1: Literature searching criterion.

## Abbreviations

3D	Three-dimensional
API	Active pharmaceutical ingredient
ASTM	American Society for Testing and Materials
CAD	Computer-aided design
CDI	Chewing Difficulty Index
DLP	Digital light processing
EMA	European Medicines Agency
EP	European Pharmacopoeia
FDA	Food and Drug Administration
FDM	Fused deposition modelling
GI	Gastrointestinal
GMP	Good Manufacturing Practices
Kgf	kilogram-force
Kp	Kilopond
MSUD	Maple Syrup Urine Disease
N	Newton
PIP	Paediatric Investigation Plan
PolyPrintlets	Multi-drug dosage forms obtained by 3D printing
Printlets	3D printed dosage forms
Scu	Strong–Cobb Units
SLS	Selective laser sintering
SSE	Semi-solid extrusion
USP	United States Pharmacopoeia
