# Supplementary material for: Innovations in Chewable Formulations: The Novelty and Applications of 3D Printing in Drug Product Design
Source: Pharmaceutics. 2022 Aug 18;14(8):1732. doi: 10.3390/pharmaceutics14081732 (PMC9412656; doi:10.3390/pharmaceutics14081732)
Supplement: Supplementary file 1 [file pharmaceutics-14-01732-s001.zip › pharmaceutics-1796058-supplementary.pdf]

Literature searching criterion

A literature search was performed using Web of Science and Google Scholar. The keywords "chewable dosage form$" and "chewable formulation$" were used to find general information about the topic, and the relevant articles on chewable formulations were selected. Subsequently, the keywords "medicated chewing gum$", "lozenge$" and "chewable tablet$" were used to search for specific references relevant to the three types of chewable formulations and gather information on formulation considerations and manufacturing methods. The keywords "medicated chewing gum$ manufacturing", "lozenge$ manufacturing" and "chewable tablet$ manufacturing" were employed to collect data on conventional manufacturing methods.

In the 3D printing section, the most relevant articles on 3D printing and its use for the preparation of chewable formulations were screened. The keywords "semisolid extrusion", "chewable tablet$", "gummy", "gel$", "hydrogel$" were selected. In the veterinary applications section, the keywords "semisolid extrusion veterinary", "3D printing veterinary", "3D printing veterinary application$" were employed.

The EMA, FDA, CIMA (Spanish Government Drug Information Centre) and EMC (Electronic Medicines Compendium) websites were used to search for marketed chewable tablets marketed for use in humans and animals. The advance search option was used to apply the "dosage form" filter, wherein only the marketed chewable tablets only were selected.
